# Ethyl 4-(5-chloro-3-methyl-1-phenyl-1*H*-pyrazol-4-yl)-6-methyl-2-oxo-1,2,3,4-tetra­hydro­pyrimidine-5-carboxyl­ate

**DOI:** 10.1107/S1600536809016365

**Published:** 2009-05-07

**Authors:** Hoong-Kun Fun, Chin Sing Yeap, K. V. Sujith, B. Kalluraya

**Affiliations:** a X-ray Crystallography Unit, School of Physics, Universiti Sains Malaysia, 11800 USM, Penang, Malaysia; b Department of Studies in Chemistry, Mangalore University, Mangalagangotri, Mangalore 574 199, India

## Abstract

In the title compound, C_18_H_19_ClN_4_O_3_, the dihydro­pyrimidin­one ring adopts a flattened boat conformation. The dihedral angle between the phenyl and pyrazole rings is 43.39 (6)°. An intra­molecular C—H⋯O contact generates an *S*(8) ring motif that stabilizes the mol­ecular conformation and precludes the carbonyl O atom of the ester group from forming inter­molecular inter­actions. Mol­ecules are linked into centrosymmetric dimers by pairs of N—H⋯O hydrogen bonds and the dimers are linked into infinite chains along [101] by N—H⋯N hydrogen bonds.

## Related literature

For medicinal applications of pyrimidinone derivatives, see: Atwal (1990 ▶); Desai *et al.* (2006 ▶); Wipf & Cunningham (1995 ▶); Bedia *et al.* (2006 ▶). For a related structure, see: Fun *et al.* (2009 ▶). For hydrogen-bond motifs, see: Bernstein *et al.* (1995 ▶). For the stability of the temperature controller used for the data collection, see: Cosier & Glazer (1986 ▶). For reference structural data, see: Allen *et al.* (1987 ▶).
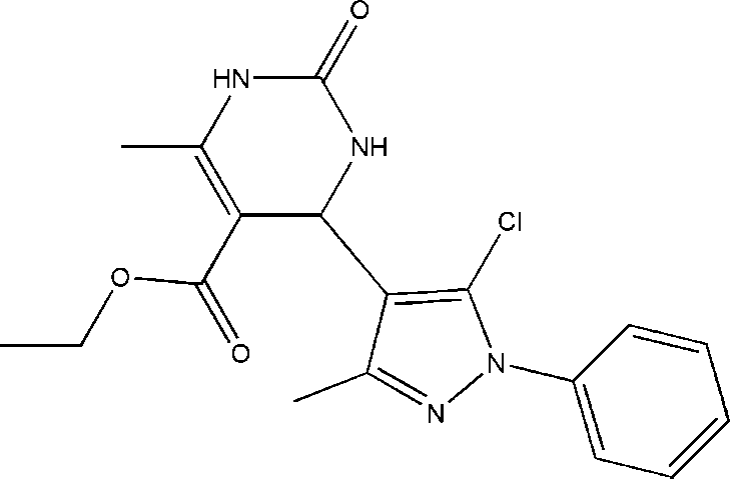


## Experimental

### 

#### Crystal data

C_18_H_19_ClN_4_O_3_
*M* *_r_* = 374.82
Triclinic, 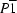

*a* = 7.9083 (2) Å
*b* = 10.2600 (2) Å
*c* = 10.9075 (3) Å
α = 93.394 (1)°
β = 99.379 (1)°
γ = 90.203 (1)°
*V* = 871.58 (4) Å^3^
*Z* = 2
Mo *K*α radiation
μ = 0.25 mm^−1^
*T* = 110 K
0.56 × 0.26 × 0.21 mm

#### Data collection

Bruker SMART APEXII CCD area-detector diffractometer
Absorption correction: multi-scan (**SADABS**; Bruker, 2005 ▶) *T*_min_ = 0.875, *T*_max_ = 0.950
18224 measured reflections
5048 independent reflections
4355 reflections with *I* > 2σ(*I*)
*R*_int_ = 0.026

#### Refinement

*R*[*F* ^2^ > 2σ(*F* ^2^)] = 0.036
*wR*(*F* ^2^) = 0.099
*S* = 1.05
5048 reflections
257 parameters
H atoms treated by a mixture of independent and constrained refinement
Δρ_max_ = 0.48 e Å^−3^
Δρ_min_ = −0.30 e Å^−3^

### 

Data collection: *APEX2* (Bruker, 2005 ▶); cell refinement: *SAINT* (Bruker, 2005 ▶); data reduction: *SAINT*; program(s) used to solve structure: *SHELXTL* (Sheldrick, 2008 ▶); program(s) used to refine structure: *SHELXTL*; molecular graphics: *SHELXTL*; software used to prepare material for publication: *SHELXTL* and *PLATON* (Spek, 2009 ▶).

## Supplementary Material

Crystal structure: contains datablocks global, I. DOI: 10.1107/S1600536809016365/tk2442sup1.cif

Structure factors: contains datablocks I. DOI: 10.1107/S1600536809016365/tk2442Isup2.hkl

Additional supplementary materials:  crystallographic information; 3D view; checkCIF report

## Figures and Tables

**Table 1:** Hydrogen-bond geometry (Å, °)

*D*—H⋯*A*	*D*—H	H⋯*A*	*D*⋯*A*	*D*—H⋯*A*
N3—H1N3⋯N2^i^	0.863 (16)	2.233 (16)	3.0601 (14)	160.7 (14)
N4—H1N4⋯O1^ii^	0.882 (17)	1.960 (17)	2.8418 (13)	176.8 (17)
C18—H18*C*⋯O3	0.96	2.59	3.2850 (15)	129

Symmetry codes: (i) 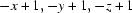; (ii) 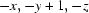.

**Fig. 1.**
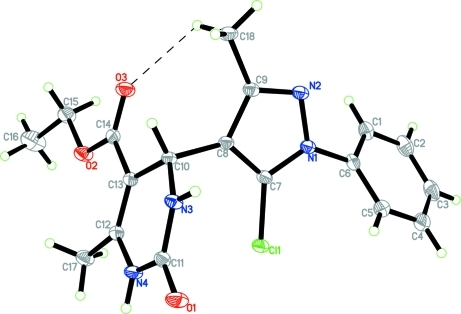
The molecular structure of (I) with atom labels and 50% probability ellipsoids for non-H atoms. An intramolecular C—H···O contact is shown as dashed lines.

**Fig. 2.**
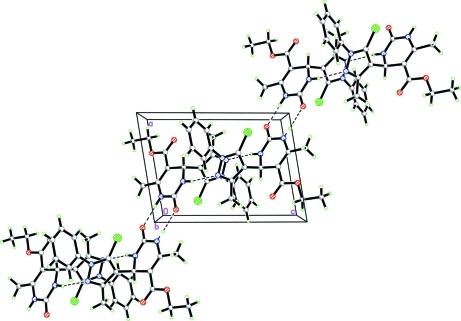
A view of the crystal packing in (I), showing an infinite chain along the [101] direction. Intermolecular hydrogen bonds are shown as dashed lines.

## supplementary crystallographic information

### Comment

Pyrimidinones have drawn widespread attention due to their medicinal applications (Atwal, 1990). A variety of dihydropyrimidinone derivatives have been screened for anti-hypertension (Desai *et al.*, 2006), anti-bacterial and anti-carcinogenic (Wipf & Cunningham, 1995), and anti-tuberculosis activity (Bedia *et al.*, 2006). Prompted by these observations and in continuation of our work in this area, herein we report the crystal structure of the title compound, (I).

In (I), Fig. 1, the dihydropyrimidinone ring adopts a flattened boat conformation. The dihedral angle between the phenyl ring and the pyrazole ring is 43.39 (6)°. Bond lengths and angles are within normal ranges and comparable to a related structure (Fun *et al.*, 2009). An intramolecular C18—H18C···O3 contact generates a *S*(8) ring motif (Bernstein *et al.*, 1995) which stabilises the molecular conformation and precludes the O3 atom from forming intermolecular contacts. The molecules are linked into centrosymmetric dimers by N—H···O hydrogen bonds (Table 1). The dimers are further linked into infinite chains along [101] by N—H···N hydrogen bonds (Fig. 2).

### Experimental

Compound (I) was obtained by refluxing a mixture of 1-phenyl-3-methyl-5-chloropyrazole-4-aldehyde (0.01 mol), ethyl acetoacetate (0.015 mol) and urea (0.01 mol) in ethanol (25 ml). The excess alcohol was removed under reduced pressure. After cooling the reaction mixture to room temperature, the contents were poured into ice-cold water (100 ml). The solid mass separated was collected by filtration and dried. Crystals were obtained from ethanol by slow evaporation (Yield 59%).

### Refinement

The N-bound H atoms were located in a difference Fourier map and refined freely. The remaining H atoms were positioned geometrically and refined using a riding model with C—H = 0.93–0.98 Å, and with U_iso_(H) = 1.2 or 1.5 U_eq_(C). A rotating-group model was applied for the methyl groups.

### Crystal data

**Table:**

C_18_H_19_ClN_4_O_3_	*Z* = 2
*M**_r_* = 374.82	*F*(000) = 392
Triclinic, *P*1	*D*_x_ = 1.428 Mg m^−^^3^
Hall symbol: -P 1	Mo *K*α radiation, λ = 0.71073 Å
*a* = 7.9083 (2) Å	Cell parameters from 9944 reflections
*b* = 10.2600 (2) Å	θ = 2.6–35.8°
*c* = 10.9075 (3) Å	µ = 0.25 mm^−^^1^
α = 93.394 (1)°	*T* = 110 K
β = 99.379 (1)°	Block, colourless
γ = 90.203 (1)°	0.56 × 0.26 × 0.21 mm
*V* = 871.58 (4) Å^3^	

### Data collection

**Table:**

Bruker SMART APEXII CCD area-detector diffractometer	5048 independent reflections
Radiation source: fine-focus sealed tube	4355 reflections with *I* > 2σ(*I*)
graphite	*R*_int_ = 0.026
φ and ω scans	θ_max_ = 30.0°, θ_min_ = 1.9°
Absorption correction: multi-scan (*SADABS*; Bruker, 2005)	*h* = −11→11
*T*_min_ = 0.875, *T*_max_ = 0.950	*k* = −14→14
18224 measured reflections	*l* = −15→15

### Refinement

**Table:**

Refinement on *F*^2^	Primary atom site location: structure-invariant direct methods
Least-squares matrix: full	Secondary atom site location: difference Fourier map
*R*[*F*^2^ > 2σ(*F*^2^)] = 0.036	Hydrogen site location: inferred from neighbouring sites
*wR*(*F*^2^) = 0.099	H atoms treated by a mixture of independent and constrained refinement
*S* = 1.05	*w* = 1/[σ^2^(*F*_o_^2^) + (0.0515*P*)^2^ + 0.3283*P*] where *P* = (*F*_o_^2^ + 2*F*_c_^2^)/3
5048 reflections	(Δ/σ)_max_ < 0.001
257 parameters	Δρ_max_ = 0.48 e Å^−^^3^
0 restraints	Δρ_min_ = −0.30 e Å^−^^3^

### Special details

**Table:**

Experimental. The crystal was placed in the cold stream of an Oxford Cyrosystems Cobra open-flow nitrogen cryostat (Cosier & Glazer, 1986) operating at 110.0 (1) K.
Geometry. All esds (except the esd in the dihedral angle between two l.s. planes) are estimated using the full covariance matrix. The cell esds are taken into account individually in the estimation of esds in distances, angles and torsion angles; correlations between esds in cell parameters are only used when they are defined by crystal symmetry. An approximate (isotropic) treatment of cell esds is used for estimating esds involving l.s. planes.
Refinement. Refinement of F^2^ against ALL reflections. The weighted R-factor wR and goodness of fit S are based on F^2^, conventional R-factors R are based on F, with F set to zero for negative F^2^. The threshold expression of F^2^ > 2sigma(F^2^) is used only for calculating R-factors(gt) etc. and is not relevant to the choice of reflections for refinement. R-factors based on F^2^ are statistically about twice as large as those based on F, and R- factors based on ALL data will be even larger.

### Fractional atomic coordinates and isotropic or equivalent isotropic displacement parameters (Å^2^)

**Table:**

	*x*	*y*	*z*	*U*_iso_*/*U*_eq_	
Cl1	0.16502 (4)	0.27288 (3)	0.28593 (3)	0.01867 (8)	
O1	0.09492 (11)	0.60474 (9)	0.13406 (8)	0.01980 (18)	
O2	0.64558 (11)	0.20298 (9)	0.00629 (8)	0.02096 (19)	
O3	0.77714 (11)	0.29869 (9)	0.18745 (8)	0.02079 (19)	
N1	0.40982 (13)	0.25693 (10)	0.48878 (9)	0.01486 (19)	
N2	0.57439 (13)	0.29637 (10)	0.53670 (9)	0.01546 (19)	
N3	0.36526 (13)	0.54573 (10)	0.21278 (9)	0.01470 (19)	
N4	0.21476 (13)	0.43462 (10)	0.03761 (9)	0.0162 (2)	
C1	0.41198 (18)	0.06888 (12)	0.61334 (11)	0.0202 (2)	
H1A	0.5286	0.0600	0.6116	0.024*	
C2	0.3256 (2)	−0.01924 (13)	0.67359 (12)	0.0253 (3)	
H2A	0.3844	−0.0884	0.7115	0.030*	
C3	0.1527 (2)	−0.00483 (14)	0.67768 (12)	0.0265 (3)	
H3A	0.0951	−0.0654	0.7165	0.032*	
C4	0.06518 (18)	0.09986 (14)	0.62404 (12)	0.0254 (3)	
H4A	−0.0503	0.1106	0.6289	0.030*	
C5	0.14923 (16)	0.18874 (13)	0.56301 (11)	0.0202 (2)	
H5A	0.0910	0.2594	0.5276	0.024*	
C6	0.32146 (16)	0.17084 (11)	0.55550 (10)	0.0161 (2)	
C7	0.36219 (14)	0.30274 (11)	0.37349 (10)	0.0143 (2)	
C8	0.49610 (14)	0.37464 (11)	0.34481 (10)	0.0132 (2)	
C9	0.62520 (14)	0.36737 (11)	0.45051 (10)	0.0144 (2)	
C10	0.50187 (14)	0.44856 (11)	0.22960 (10)	0.0135 (2)	
H10A	0.6117	0.4960	0.2419	0.016*	
C11	0.21883 (15)	0.53209 (11)	0.13033 (10)	0.0150 (2)	
C12	0.35013 (15)	0.35289 (11)	0.02438 (10)	0.0146 (2)	
C13	0.49312 (14)	0.35937 (11)	0.11231 (10)	0.0140 (2)	
C14	0.65113 (15)	0.28596 (11)	0.10717 (11)	0.0155 (2)	
C15	0.80143 (17)	0.13146 (13)	−0.00265 (12)	0.0223 (3)	
H15A	0.8243	0.0716	0.0635	0.027*	
H15B	0.8983	0.1912	0.0039	0.027*	
C16	0.7726 (2)	0.05757 (18)	−0.12749 (15)	0.0387 (4)	
H16A	0.8746	0.0112	−0.1390	0.058*	
H16B	0.7456	0.1177	−0.1918	0.058*	
H16C	0.6793	−0.0034	−0.1314	0.058*	
C17	0.31577 (17)	0.26349 (13)	−0.09159 (12)	0.0201 (2)	
H17A	0.207 (3)	0.2792 (18)	−0.1382 (18)	0.034 (5)*	
H17B	0.316 (3)	0.175 (2)	−0.0718 (18)	0.038 (5)*	
H17C	0.398 (2)	0.2794 (18)	−0.1447 (17)	0.034 (5)*	
C18	0.79965 (15)	0.42889 (12)	0.47221 (12)	0.0187 (2)	
H18A	0.8530	0.4188	0.5566	0.028*	
H18B	0.7902	0.5201	0.4575	0.028*	
H18C	0.8679	0.3874	0.4164	0.028*	
H1N3	0.367 (2)	0.6038 (15)	0.2734 (15)	0.016 (4)*	
H1N4	0.120 (2)	0.4247 (17)	−0.0173 (17)	0.026 (4)*	

### Atomic displacement parameters (Å^2^)

**Table:**

	*U*^11^	*U*^22^	*U*^33^	*U*^12^	*U*^13^	*U*^23^
Cl1	0.01327 (13)	0.02560 (15)	0.01588 (13)	−0.00443 (10)	−0.00218 (9)	0.00376 (10)
O1	0.0153 (4)	0.0211 (4)	0.0210 (4)	0.0060 (3)	−0.0022 (3)	−0.0012 (3)
O2	0.0159 (4)	0.0277 (5)	0.0182 (4)	0.0091 (3)	0.0006 (3)	−0.0024 (3)
O3	0.0145 (4)	0.0270 (5)	0.0195 (4)	0.0048 (3)	−0.0011 (3)	0.0009 (3)
N1	0.0128 (4)	0.0176 (4)	0.0136 (4)	−0.0006 (3)	−0.0002 (3)	0.0026 (3)
N2	0.0128 (4)	0.0175 (4)	0.0149 (4)	0.0011 (3)	−0.0010 (3)	0.0006 (3)
N3	0.0139 (4)	0.0149 (4)	0.0140 (4)	0.0030 (3)	−0.0013 (3)	0.0004 (3)
N4	0.0122 (5)	0.0200 (5)	0.0149 (4)	0.0032 (4)	−0.0019 (4)	−0.0010 (4)
C1	0.0254 (6)	0.0195 (5)	0.0158 (5)	0.0032 (5)	0.0035 (4)	0.0025 (4)
C2	0.0378 (8)	0.0200 (6)	0.0182 (6)	0.0003 (5)	0.0033 (5)	0.0047 (5)
C3	0.0361 (8)	0.0271 (6)	0.0160 (6)	−0.0124 (6)	0.0031 (5)	0.0039 (5)
C4	0.0224 (6)	0.0350 (7)	0.0190 (6)	−0.0072 (5)	0.0037 (5)	0.0033 (5)
C5	0.0192 (6)	0.0236 (6)	0.0179 (5)	−0.0001 (4)	0.0027 (4)	0.0033 (4)
C6	0.0201 (6)	0.0164 (5)	0.0115 (5)	−0.0015 (4)	0.0021 (4)	0.0010 (4)
C7	0.0132 (5)	0.0161 (5)	0.0129 (5)	0.0009 (4)	−0.0002 (4)	0.0014 (4)
C8	0.0110 (5)	0.0147 (5)	0.0133 (5)	0.0017 (4)	0.0004 (4)	0.0009 (4)
C9	0.0125 (5)	0.0150 (5)	0.0147 (5)	0.0022 (4)	−0.0002 (4)	−0.0004 (4)
C10	0.0100 (5)	0.0159 (5)	0.0141 (5)	0.0011 (4)	0.0000 (4)	0.0026 (4)
C11	0.0145 (5)	0.0155 (5)	0.0147 (5)	0.0006 (4)	0.0006 (4)	0.0030 (4)
C12	0.0132 (5)	0.0168 (5)	0.0139 (5)	0.0017 (4)	0.0023 (4)	0.0020 (4)
C13	0.0125 (5)	0.0164 (5)	0.0132 (5)	0.0019 (4)	0.0017 (4)	0.0021 (4)
C14	0.0142 (5)	0.0180 (5)	0.0148 (5)	0.0018 (4)	0.0027 (4)	0.0044 (4)
C15	0.0179 (6)	0.0280 (6)	0.0212 (6)	0.0107 (5)	0.0032 (5)	0.0016 (5)
C16	0.0343 (8)	0.0504 (10)	0.0292 (8)	0.0220 (7)	0.0025 (6)	−0.0105 (7)
C17	0.0182 (6)	0.0246 (6)	0.0157 (5)	0.0029 (5)	−0.0008 (4)	−0.0031 (4)
C18	0.0120 (5)	0.0226 (6)	0.0200 (6)	0.0000 (4)	−0.0010 (4)	0.0007 (4)

### Geometric parameters (Å, °)

**Table:**

Cl1—C7	1.7070 (12)	C5—C6	1.3899 (17)
O1—C11	1.2378 (14)	C5—H5A	0.9300
O2—C14	1.3456 (14)	C7—C8	1.3760 (16)
O2—C15	1.4505 (14)	C8—C9	1.4158 (15)
O3—C14	1.2157 (14)	C8—C10	1.5125 (15)
N1—C7	1.3641 (14)	C9—C18	1.4924 (16)
N1—N2	1.3712 (13)	C10—C13	1.5202 (15)
N1—C6	1.4268 (15)	C10—H10A	0.9800
N2—C9	1.3334 (15)	C12—C13	1.3570 (15)
N3—C11	1.3468 (14)	C12—C17	1.5047 (16)
N3—C10	1.4695 (14)	C13—C14	1.4685 (15)
N3—H1N3	0.862 (16)	C15—C16	1.5020 (19)
N4—C11	1.3760 (15)	C15—H15A	0.9700
N4—C12	1.3828 (14)	C15—H15B	0.9700
N4—H1N4	0.883 (18)	C16—H16A	0.9600
C1—C2	1.3886 (18)	C16—H16B	0.9600
C1—C6	1.3932 (17)	C16—H16C	0.9600
C1—H1A	0.9300	C17—H17A	0.95 (2)
C2—C3	1.383 (2)	C17—H17B	0.95 (2)
C2—H2A	0.9300	C17—H17C	0.956 (19)
C3—C4	1.387 (2)	C18—H18A	0.9600
C3—H3A	0.9300	C18—H18B	0.9600
C4—C5	1.3887 (18)	C18—H18C	0.9600
C4—H4A	0.9300		
			
C14—O2—C15	115.79 (9)	C8—C10—C13	112.92 (9)
C7—N1—N2	110.16 (9)	N3—C10—H10A	107.5
C7—N1—C6	130.02 (10)	C8—C10—H10A	107.5
N2—N1—C6	119.60 (9)	C13—C10—H10A	107.5
C9—N2—N1	105.46 (9)	O1—C11—N3	122.70 (10)
C11—N3—C10	125.12 (9)	O1—C11—N4	120.82 (10)
C11—N3—H1N3	117.1 (10)	N3—C11—N4	116.45 (10)
C10—N3—H1N3	115.4 (10)	C13—C12—N4	119.61 (10)
C11—N4—C12	124.24 (10)	C13—C12—C17	127.57 (10)
C11—N4—H1N4	117.5 (11)	N4—C12—C17	112.82 (10)
C12—N4—H1N4	118.3 (11)	C12—C13—C14	125.95 (10)
C2—C1—C6	119.05 (12)	C12—C13—C10	120.88 (10)
C2—C1—H1A	120.5	C14—C13—C10	113.16 (9)
C6—C1—H1A	120.5	O3—C14—O2	122.46 (11)
C3—C2—C1	120.45 (12)	O3—C14—C13	122.92 (11)
C3—C2—H2A	119.8	O2—C14—C13	114.63 (10)
C1—C2—H2A	119.8	O2—C15—C16	106.46 (10)
C2—C3—C4	120.08 (13)	O2—C15—H15A	110.4
C2—C3—H3A	120.0	C16—C15—H15A	110.4
C4—C3—H3A	120.0	O2—C15—H15B	110.4
C3—C4—C5	120.28 (13)	C16—C15—H15B	110.4
C3—C4—H4A	119.9	H15A—C15—H15B	108.6
C5—C4—H4A	119.9	C15—C16—H16A	109.5
C4—C5—C6	119.22 (12)	C15—C16—H16B	109.5
C4—C5—H5A	120.4	H16A—C16—H16B	109.5
C6—C5—H5A	120.4	C15—C16—H16C	109.5
C5—C6—C1	120.82 (12)	H16A—C16—H16C	109.5
C5—C6—N1	120.71 (11)	H16B—C16—H16C	109.5
C1—C6—N1	118.47 (11)	C12—C17—H17A	111.0 (11)
N1—C7—C8	108.70 (10)	C12—C17—H17B	111.1 (12)
N1—C7—Cl1	123.11 (9)	H17A—C17—H17B	106.9 (16)
C8—C7—Cl1	128.18 (9)	C12—C17—H17C	110.3 (11)
C7—C8—C9	103.79 (10)	H17A—C17—H17C	106.4 (16)
C7—C8—C10	128.17 (10)	H17B—C17—H17C	111.0 (17)
C9—C8—C10	128.00 (10)	C9—C18—H18A	109.5
N2—C9—C8	111.88 (10)	C9—C18—H18B	109.5
N2—C9—C18	120.43 (10)	H18A—C18—H18B	109.5
C8—C9—C18	127.69 (11)	C9—C18—H18C	109.5
N3—C10—C8	111.17 (9)	H18A—C18—H18C	109.5
N3—C10—C13	110.10 (9)	H18B—C18—H18C	109.5
			
C7—N1—N2—C9	0.68 (12)	C11—N3—C10—C8	−103.04 (12)
C6—N1—N2—C9	175.80 (10)	C11—N3—C10—C13	22.87 (15)
C6—C1—C2—C3	−0.95 (19)	C7—C8—C10—N3	57.33 (15)
C1—C2—C3—C4	−1.6 (2)	C9—C8—C10—N3	−120.05 (12)
C2—C3—C4—C5	1.8 (2)	C7—C8—C10—C13	−67.00 (15)
C3—C4—C5—C6	0.54 (19)	C9—C8—C10—C13	115.62 (12)
C4—C5—C6—C1	−3.09 (18)	C10—N3—C11—O1	166.80 (11)
C4—C5—C6—N1	177.20 (11)	C10—N3—C11—N4	−15.22 (16)
C2—C1—C6—C5	3.29 (18)	C12—N4—C11—O1	177.43 (11)
C2—C1—C6—N1	−176.99 (11)	C12—N4—C11—N3	−0.59 (17)
C7—N1—C6—C5	−46.54 (17)	C11—N4—C12—C13	5.61 (18)
N2—N1—C6—C5	139.45 (11)	C11—N4—C12—C17	−174.82 (11)
C7—N1—C6—C1	133.75 (13)	N4—C12—C13—C14	−175.52 (11)
N2—N1—C6—C1	−40.27 (15)	C17—C12—C13—C14	5.0 (2)
N2—N1—C7—C8	−0.59 (13)	N4—C12—C13—C10	4.26 (17)
C6—N1—C7—C8	−175.05 (11)	C17—C12—C13—C10	−175.23 (11)
N2—N1—C7—Cl1	−179.88 (8)	N3—C10—C13—C12	−16.61 (15)
C6—N1—C7—Cl1	5.67 (17)	C8—C10—C13—C12	108.30 (12)
N1—C7—C8—C9	0.25 (12)	N3—C10—C13—C14	163.20 (9)
Cl1—C7—C8—C9	179.49 (9)	C8—C10—C13—C14	−71.89 (12)
N1—C7—C8—C10	−177.62 (10)	C15—O2—C14—O3	−1.48 (17)
Cl1—C7—C8—C10	1.62 (18)	C15—O2—C14—C13	178.58 (10)
N1—N2—C9—C8	−0.52 (12)	C12—C13—C14—O3	177.86 (12)
N1—N2—C9—C18	178.99 (10)	C10—C13—C14—O3	−1.93 (17)
C7—C8—C9—N2	0.18 (13)	C12—C13—C14—O2	−2.20 (17)
C10—C8—C9—N2	178.06 (10)	C10—C13—C14—O2	178.00 (10)
C7—C8—C9—C18	−179.29 (11)	C14—O2—C15—C16	−173.84 (12)
C10—C8—C9—C18	−1.41 (19)		

### Hydrogen-bond geometry (Å, °)

**Table:**

*D*—H···*A*	*D*—H	H···*A*	*D*···*A*	*D*—H···*A*
N3—H1N3···N2^i^	0.863 (16)	2.233 (16)	3.0601 (14)	160.7 (14)
N4—H1N4···O1^ii^	0.882 (17)	1.960 (17)	2.8418 (13)	176.8 (17)
C18—H18C···O3	0.96	2.59	3.2850 (15)	129

Symmetry codes: (i) −*x*+1, −*y*+1, −*z*+1; (ii) −*x*, −*y*+1, −*z*.
